# Navigating Rehabilitation Transitions at Street Level: A Qualitative Analysis of Municipal Service Allocation to Individuals With Complex Needs

**DOI:** 10.1177/11786329241293347

**Published:** 2024-10-20

**Authors:** Maren Ekenes, Eike Wehling, Olin Oldeide

**Affiliations:** 1 Department of Physical Medicine and Rehabilitation, Haukeland University Hospital, Bergen, Norway; 2 Department of Health Promotion and Development, University of Bergen, Bergen, Norway; 3 Department of Biological and Medical Psychology, University of Bergen, Bergen, Norway

**Keywords:** Complex needs, rehabilitation trajectory, individually tailored services, location, case workers, acquired brain injury (ABI), qualitative research

## Abstract

The transition of patients with complex needs from hospital to municipal rehabilitation following moderate and severe brain injury is challenging. This qualitative study explored the municipal service allocation processes within such transitions. The caseworkers’ comprehensive task of combining patients’ preferences and needs, healthcare providers’ recommendations and municipal guidelines and service allocation were analysed. Data comprised of patients’ health records, meeting observations and semi-structured interviews with municipal staff, patients and next of kin. Results demonstrated that the issue of most concern was the location of where the patient was to continue municipal rehabilitation. Municipal caseworkers gathered extensive information, including recommendations from healthcare providers and preferences of patients and next of kin. These were frequently in contrast to the municipal guidelines’ requirements and the services’ organisational structure. The discrepancies led to tension, which was difficult to manoeuvre. This study indicates that incorporating individually tailored services into the daily service allocation practice can be demanding and even dilemmatic. The designated focus on the transition of patients with complex rehabilitation needs gives insights into how service allocation, user involvement and coordination policies are acted out in practice and may directly influence rehabilitation trajectories.

## Introduction

Globally, there is a growing population of survivors of moderate and severe brain injury (ABI) who experience complex cognitive, emotional, behavioural and physical changes.^1,2^ These patients’ rehabilitation, understood as a person-centred, collaborative process of multiple interventions to optimise function and reduce disability,^3^ is commonly initiated in hospitals and continued at the municipal level.^1,4-6^ Multiple professions can be included in the rehabilitation process, such as physicians, nurses, occupational and physiotherapists, social workers, speech and language therapists and neuropsychologists and others.^7^ The transition from hospital to municipal rehabilitation is recognised as important because crucial information is transferred and decisions on further rehabilitation services are to be made, impacting individual rehabilitation trajectories.^1,8^ These processes are described as particularly vulnerable and complex as they involve multiple stakeholders crossing organisational and professional structures.^9^ Previous research has revealed common challenges during this transition process to include lack of coordination between levels (ie, hospital and municipality), communication with loss or delay, varying agreement and approach of risks for the patient and increased risk of hospital readmission.^10-12^

To enhance the quality of these processes, particular importance is placed on the necessity of coordinating efforts between service providers and the integration of the user’s preferences and needs.^13-18^ The pursuit of user involvement is expressed in mottos such as ‘no decision about me, without me’.^19-21^ The goal is to include the user’s perspective on what is important and to empower patients and next of kin to take part in negotiations and decisions regarding their care trajectory, regardless of impairment and healthcare settings.^22-24^ Such an approach is believed to create trust, while actively shaping individually tailored, coordinated services.^22,24,25^ Yet, patients and next of kin have described transition processes as stressful and filled with uncertainty.^6,26^ A lack of sufficient involvement in discussions and decision-making has been reported.^27,28^ Patients and next of kin express difficulty in navigating complex and fragmented services as well as expressing a high service access burden.^29^

Facing increasing resource demands, many Western countries have implemented healthcare reforms to simultaneously reduce costs and enhance care coordination of healthcare services.^30-33^ Increased focus on care transitions has been found following these reforms.^34^ In Norway, where this study is situated, the ‘Coordination Reform’ was implemented to enhance the coordination of services between hospitals and municipalities, promote earlier hospital discharge and greater municipal responsibility in rehabilitation processes.^32,33,35^ Hospitals were given the authority to decide when the patient was ready to be discharged. Municipalities were authorised to decide which services to offer following the hospital stay.^35,36^ Economic, legal, organisational, and healthcare measures were implemented to enhance coordination between the 2 levels of healthcare services.^32,33^ Simultaneously, purchaser-provider models (PPM) have influenced the organisation of Norwegian municipal healthcare services, whereby distinctions are made between those deciding service allocation (separate municipal allocation offices with designated caseworkers) and the providers of such services (healthcare providers within the municipality).^37^ At the allocation offices, caseworkers are responsible for managing equal access to the collective benefits of municipal healthcare services, while also ensuring correct case proceedings and allocating individually tailored services.^37,38^ As such, municipal caseworkers become the operative link between policymakers and patients, and thus play a vital role as street-level bureaucrats in the implementation of healthcare policy.^39^ In practice, their role may become comprehensive and dilemmatic when contrasting requirements, such as available services and users’ preferences and needs, clash with one another.^38-41^ Foster et al,^42^ suggest studying care transitions based on street-level perspectives, to gain a deeper understanding of how organisations and their staff work and adapt to the challenges they face. This deepened understanding would provide insights into how policy is acted out in real-life situations and is suitable to inform measures both in policy and in practice.^43^

To our knowledge, few studies provide in-depth analysis of ongoing decision processes of service allocation following hospital discharge.^42^ So far, challenges of service allocation experienced by municipal caseworkers have mainly been examined in elderly care.^14,41^ Findings reveal that tailoring services to individual needs in accordance with the preferences and needs of service users is complex, due to resource limitations, fragmentation of services, organisational requirements and standardisation measures, as well as tight time-frames for decision-making.^14,41,44,45^ Service allocation in care transitions of the growing population of individuals with moderate and severe ABI, is still scarcely investigated, and knowledge of which challenges occur and how they are dealt with in real-life rehabilitation transitions needs further examination.^14,26,46,47^

To contribute to further insights into navigating transitions of rehabilitation trajectories, this study applies a street-level perspective to illuminate challenges of service allocation. By applying this perspective, the study explores on-the-ground realities of health care provision which may differ from intentions and ‘best-practice’ descriptions displayed in guidelines and policies.^43^ Hence, to further inform both policy development and practice, this study aims to illuminate how challenges of service allocation are managed in practice, as service allocation decisions must be made while competing considerations may be present.^39,42,43^ The research question is: During the transition from hospital to municipal care for patients with moderate and severe brain injury, how do challenges arise in the service allocation process at the municipal level?

## Material and Methods

### Study design and context

The study, conducted in Norway, is a part of a qualitative case study to provide an in-depth exploration of rehabilitation transition and municipal decisions of service allocation.^48,49^ The study was approved by the Norwegian Centre for Research Data (NSD) and the hospital data protection officer.

### Participants and recruitment

Trajectories of 10 patients were included in the study, including data from patients, next of kin, health care staff, caseworkers and municipal leaders. Patients aged 18 to 80 years, staying at one hospital due to moderate or severe ABI, and receiving rehabilitation by a multidisciplinary team were recruited. The recruitment was carried out by a physician on behalf of the researchers.

The inclusion criteria were:

- Patient admitted to the hospital rehabilitation unit following a moderate to severe brain injury.
- Patients in need of 4 or more professions involved in further municipal rehabilitation following discharge.

There were no explicit exclusion criteria. Written informed consent was obtained from patients and next of kin/legal guardians, with the option to withdraw at any time, prior to inclusion. For patients unable to consent due to the severeness of their injury, written informed consent was obtained from their legal guardian. Patients unable to provide informed consent are often excluded from studies for ethical reasons.^50^ In this study, ethical authorities deemed the participation risk low and insights valuable, so lack of consent ability by patients was not an exclusion criterion. Due to ethical restrictions, demographic information on participants is anonymised and not provided in this study.

Both hospital and municipal managers approved for the organisations to participate in the study. To examine transitions at the municipal level, 2 municipalities were recruited to the study, creating the opportunity to combine data from different contexts.^49^ The municipal staff in the 2 municipalities (managers, caseworkers and care providers) were recruited successively (snowball sampling), as they took part in the transition process.^51^ Prior to all involvement, participating staff gave their informed consent in writing or verbal, depending on the degree of participation in the study. Verbal consent was given in front of a third party. All contact included the information of voluntary participation, the opportunity to withdraw consent at any time and that participation would be treated anonymously.

#### Data collection

Our data includes extractions from patients’ health records, for example, meeting notes, written correspondence and service applications, semi-structured interviews and observation of transition meetings between hospital staff and municipal staff, patients and next of kin. We chose a triangulation of sources to create diverse and rich data material for in-depth analysis, suitable to illuminate how challenges of service allocation arise and are met in real-life care trajectories.^49,52^

Transition meetings between the hospital rehabilitation unit, municipalities and next of kin/patients were observed prior to discharge (8). To ensure rigour, an observation guide was developed focusing on how municipal service allocation was addressed and managed in the meetings. Observation notes were made. The observations were done by the first author, who was presented as a researcher at the beginning of each meeting.

Post-discharge, documents from patient health records were extracted by the hospital, the municipalities and patients/next of kin. The documents consisted of meeting minutes, applications, case reports, electronic correspondence between the municipalities and the hospital and decisions on service allocation.

Following discharge, semi-structured interviews were held with patients, next of kin/legal guardians, municipal case workers, managers and service providers. An interview guide, developed by the first author, provided structure for the interviews focusing on the possesses of care transition and service allocation. It contained open-ended questions to gather more detailed information of the transition processes and service allocation from the participant’s perspective. The interviews provided the opportunity to elaborate on the transition and service allocation processes, the observed meetings and available documents. Further, they provided a view through the lens of the stakeholder and thus gained further insights into the allocation processes.^49^ Interview questions concerned the individual transition process, responsibilities, roles, municipal resources, the use of decision tools and the organisation of services.

Interviews were conducted at locations, face to face or by telephone, according to the participant’s wishes. Clinicians and managers were interviewed as a group or individually, depending on their wishes. Between 9 and 14 participants were interviewed for each trajectory. In 3 of the trajectories, the caseworkers declined to be interviewed. The interviews were recorded and varied in length (30-90 minutes). Data was stored in accordance with ethical requirements in a secure research database. All data gathering was done by the first author. Combined, the data provided a ‘thick’ description of the transition and service allocation process, suited to explore the research question.^49^

### Analysis

The analysis is inspired by the 6 phases described as thematic analysis by Braun and Clarke.^53^ Phase 1 started with the first author, reassessing the gathered data. This was done to ensure familiarisation with the data and initial search for meaning and patterns. In phase 2, extractions from the written documents were chronologically ordered in flowcharts using Microsoft Visio. Short extracts from the observed meetings and interviews were added to gain a more thorough understanding of the individual transition. Together the extractions were directed towards information exchange and the process of service allocation. Health descriptions, demographic data and dates were modified to ensure anonymity. The flowcharts provided a descriptive and visual summary for further exploration, suitable to compare the individual transitions.^49,54^

A condensed summary of the most common features of the service allocation process was developed and presented to 2 experienced and central municipal staff members. They agreed with the overall account and the initial analysis. This member checking provided the participant’s perspective and an opportunity to provide feedback and address misunderstandings.^55^ This was also an essential step to strengthen reflexivity.^55^ Further, the first author identified the data concerning wishes from users and next of kin, recommendations from service providers and case workers and organised it in a table to be assessed in the interpretive analysis.

In phase 3, the authors searched and discussed patterns and themes in the data, focusing especially on challenges in the transitions. The positionality of the researchers impacts the research process. The authors provided different entry points to the analysis, combining clinical and research experience and perspectives from the fields of rehabilitation/ nursing (first author), rehabilitation/ psychology (second author) and health promotion (third author). The combining of different perspectives is in line with investigator triangulation which may limit bias and enhance reflexivity. This phase included a back-and-forth process between data and theory, in line with an abductive approach, exploring the phenomena through empirical data, relevant research and theoretical perspectives.^56^ Finally, the analysis illuminated challenges concerning the allocation of services with a street-level perspective.

In phase 4, the themes were reviewed in relation to the data developed in phase 2 and 3, consequently, the data set in its entirety, which allowed for further refining of the themes and naming of themes in phase 5. The final phase consisted of the reporting of the analysis, providing illustrative extracts capturing the essence of the 3 main themes: (1) Merging municipal guidelines and hospital recommendation, (2) Involving the patient and next of kin in the decision process, (3) Discrepancy between available services and recommendations of municipal rehabilitation providers.

## Results

In the paragraphs below, we outline the typical service allocation processes, as identified by our analysis. Further, the 3 main themes found in our analysis are expounded.

### The service allocation processes

Our analysis showed that the communication regarding further municipal rehabilitation started when the municipality, that is the service allocation offices, received information regarding a patient in need of rehabilitation. Typically, this occurred within the first week after admission to the hospital rehabilitation unit. In order to prepare for the transition from hospital to municipal rehabilitation, a first meeting was initiated several weeks or even months before discharge. Time points for both first meetings and thereafter discharge varied considerably (admission to first meeting 25-131 days after admission; first meeting to discharge 18 and 111 days). In 3 trajectories, the pattern differed; in 2 trajectories meetings were regarded to be unnecessary by the hospital and the municipality due to sufficient information exchange using electronic communication and telephone communication; in the third one a meeting was held 2 days before discharge.

Municipal caseworkers, the hospital rehabilitation team and next of kin/legal guardians attended the meetings held at the hospital. A minority of the patients attended the meetings together with their next of kin. During the meetings, the patient’s interdisciplinary hospital rehabilitation team gave a summary of the patient’s medical history, health status, current rehabilitation and goals, and roughly estimated date of discharge.

The caseworkers would ask the patients or next of kin about their preferences and needs. Based on our interpretation of the data, a main issue in the service allocation process concerned the question as to *where* the patient would receive further rehabilitation. In several trajectories, electronic communication, follow-up meetings and phone calls continued to address the matter further. Our analysis displayed that considerations regarding final decisions concerning location were linked to municipal allocation guidelines and the kind of services the location had available (eg, occupational therapy and physiotherapy), and to a lesser extent, to long-term planning or social concerns.

### Theme 1: Merging municipal guidelines and hospital recommendations

Throughout the service allocation processes, municipal caseworkers and managers stressed the importance of local guidelines for service allocation:

We have criteria for all services we assign. We have a booklet on work processes. We call it our bible. And then we have criteria for the allocation of care and care services which is a daily working tool. (Interview, manager, municipality)

Simultaneously, it was emphasised that thorough information gathering regarding the needs of the patient was important to allocate the appropriate services:

The criteria of service allocation are always the foundation for decisions. And then we’re to get information from all parties. All information is considered, what emerges from the meetings, from the electronic messages (from the hospital), perhaps from a telephone conversation that follows, what the individual wants. Everything must be included. We must address all sides of a case. That is very important. We can’t ignore any side. (Interview, caseworker)

Hospital staff emphasised the need for further municipal rehabilitation. Recommendations were quite specific regarding location and the kind of services deemed to support the best possible outcome and long-term perspective. Our analysis shows that arguments for the specific location of further rehabilitation concerned for example (1) location in geographical proximity to the patient’s home, to for example facilitate family support and joint activities, (2) the need for further in-patient rehabilitation, (3) allocation of assistants in the patient’s home or (4) direct transfer to long-term housing facilities. The latter was to reduce the number of transfers and to ensure knowledge transfer from the hospital to the municipal healthcare providers responsible for the anticipated long-term care of the patient.

Analysis of meetings and documents displayed that during the joint meetings, the municipal caseworkers briefly informed attendees about the options regarding location, varying between a short-term stay in a municipal rehabilitation unit, discharge to the home or living in a nursing home in close proximity to the family, the latter without designated rehabilitation services for the relevant age group. They underlined that decisions concerning location would not be made within the meeting. Recommendations and preferences regarding location were reported by caseworkers in internal case descriptions:

The interdisciplinary hospital team suggests that the patient is to be allocated a long-term rehabilitation stay at the local nursing home geographically close to the family. This is to enforce stability for the patient over time, to ensure a good transfer of knowledge to personnel (with long-term responsibility for care), and to place the patient in a familiar environment close to the family. This is also what the family wants. (Case description, caseworker)

Our analysis revealed that municipal caseworkers informed already in the initial meetings, that recommendations could not be met, due to guideline criteria. These included that certain services could only be allotted at a later point in time (long-term nursing facilities), that the patient needed to meet certain age criteria, or that the municipal structure was not designed to relocate services between different service-providing units (eg, occupational therapy and physiotherapy to local nursing homes). The most common criterion that presented challenges was that the patients did not meet the age criteria for a specific location:

Informed during the meeting that patients in this age group and who need rehabilitation are allocated a short term stay at the specific rehabilitation unit. The issue has been discussed with the decision-making unit, and it is confirmed that this is the right unit for the patient. (Though in contradiction to the recommendation of the hospital and preferences of next of kin). (Health care record, case note, caseworker)

When the municipal location options were presented, our analysis showed that this repeatedly led to tensions due to the discrepancy between the hospitals’ recommendations, preferences of patients and next of kin and available options of municipal services. Municipal solutions could be disputed openly in meetings, by telephone or in electronic communication, by hospital staff:

‘Based on our experience, this (the offered municipal solution) will cause the patient to be discharged to a facility lacking necessary rehabilitation personnel. We regard this as professionally unjustifiable. On this ground we await confirmation of the patient receiving municipal in-patient rehabilitation before being discharged from the hospital rehabilitation unit’. (Health care record, telephone memo between hospital staff and municipal allocation office)

In situations with high tensions, caseworkers could leave the joint meetings to contact their managers to discuss whether there were feasible alternatives. Yet, always confirming the necessity to adhere to the guidelines upon return. Furthermore, in several transitions, back-and-forth communication with the hospital led to continuing investigation by the caseworker:

I said I would raise the question again to my manager. To see if the preferred service solution was possible to allocate (after all). I called the centre (again), and I received the response that it was not feasible. That it was not a relevant issue. (Interview, caseworker)

The caseworkers expressed in the interviews how recommended services were difficult to allocate, but that they searched for alternatives. The attempts made by the hospital staff to find alternative solutions were viewed as problematic by the caseworkers and managers since these could raise false hopes for patients and next of kin as to what the municipality could provide:

The hospital called me. They said that they were trying to be creative. Then I said that they should not be so creative. At least they must discuss it with us (the service allocation offices) before involving the next of kin. Such suggestions (for service allocation) need to be thought through thoroughly before giving hope that something is possible. (Interview, caseworker)

### Theme 2: Involving the patient and next of kin in the decision process

According to our analysis, the caseworkers made great efforts to gather information and identify the patient’s or next of kin’s preferences and needs by directly addressing them in meetings, continuing the communication after meetings and gathering information indirectly from the hospital staff. Most patients were unable to attend meetings or to formulate their preferences and needs. Often, the next of kin seemed to have difficulties to specifically express what was important to them (the patient and/or next of kin) or what they preferred, particularly at the joint meetings with municipal caseworkers and hospital staff. In the following quote, the next of kin answered what matters were important for the patient/them after the hospital discharge:

It is difficult to answer. But I want him to come home in the end. (Meeting, next of kin)

Even though preferences and needs were difficult to define, most were concerned with the time of the discharge:

I must say, when they told me I had to continue the rehabilitation at the municipal rehabilitation unit. It really put me back. It was just that . . . when you are prepared to be at the hospital. And then suddenly you are to go to a new place. You are not well. (Interview, patient)

Our analysis further revealed that the preferences and needs of the next of kin and the patient did not always match. Hence, in some transitions, the caseworkers were confronted with a discrepancy between the patient’s preferences (eg, direct discharge to home) and what the next of kin regarded as conceivable (eg, the family’s capacity to take care of the individual at home). The question was primarily presented as a dilemma for the next of kin:

Meeting to inform on alternative housing facilities and assistance in the home. Initially the response of the partner was that she does not have the capacity to undertake the administrative tasks of taking NN home. At the end of the meeting, she stated that if the service depends on her, she needs to think a little more about whether she may still have to take on this responsibility. (Memo of follow-up meeting at municipal decision office between caseworker and next of kin)

Additionally, patients and the next of kin often expressed great uncertainty about what further rehabilitation municipal services would entail:

We wanted him to stay there (the hospital) as long as possible. So, it was a little bit . . . I was stunned. I did not know what we were going to get from the municipality. The patient confirms (words unclear due to aphasia) . . . (Interview, next of kin and patient)

Since hospital recommendations and municipal solutions were openly discussed, discrepancies were revealed, and the next of kin were left in the difficult situation of either accepting the municipality’s solutions, commonly in discrepancy with the hospital recommendations or to try and negotiate alternative options. Municipal caseworkers and municipal managers stated that the joint meetings, displaying disagreement and tension, could be unfortunate as it signalled a fragmented healthcare service. Hence, such meetings could potentially jeopardise trust and further cooperation with the next of kin and patients. As a solution, they suggested preliminary meetings between the municipality and hospital staff to clarify service options prior to the involvement of patients and next of kin:

What we want is a preliminary meeting with the hospital (without patients and next of kin). To get clarification. Then we have to appear unified when we have meetings with patients and relatives. It is a very unfortunate experience for the relatives that they must take part and see that the service is not coordinated. It is unfortunate that the next of kin do not encounter a unified health service. This has consequences for further cooperation with relatives. (Interview, municipal manager, municipal decision-making office)

In several of the transitions, caseworkers continued communication with the next of kin following meetings to resolve uncertainties, consider concrete preferences and further negotiate service solutions.

### Theme 3: Discrepancy between available services and recommendations of municipal rehabilitation providers

By analysing the allocation processes, we found that, while trying to find residency for the patient, caseworkers obtained recommendations from municipal care providers regarding further services. In more than half of the transitions, these providers reported back that they found the discharge dates to be too early, unit resources to be insufficient to meet the complex needs of patients (citation a) or specific services to be unavailable (citation b).

(a) ‘I think that many people are discharged from hospital far too early. This is also true for this patient – there is a huge difference between the hospital rehabilitation unit and this location when it comes to resources . . . There is also a discussion of when patients become very resource-intensive. Who’s going to pay? It is both finances and personnel that can be challenging’. (Interview, medical doctor municipal rehabilitation unit)
(b) ‘According to the hospital summary, the patient has sustained a very serious injury with poor prognosis in terms of improvement of functional level. No adequate communication. Must have help in all care situations. Not able to participate in setting rehabilitation goals. Further, we do not have a speech therapist at our location. Based on the available information, the patient is too poorly functioning to be able to benefit from the rehabilitation offered at our location. Hence, we recommend that a rehabilitation stay at this unit is not offered’. (Letter recommending rejection, municipal rehabilitation unit)

The municipal healthcare providers emphasised that patients would not benefit from staying in their unit and that a stay could lead to frustration in patients or next of kin. Furthermore, they stated that the patients should be able to participate in goal-setting and self-driven activities to receive rehabilitation at their facility but that such a role could not be fulfilled by several of the patients in question due to the severity of their brain injuries. Alternatively, for such recommendations, case workers would explore other possibilities, such as assembling an interprofessional team at alternative locations such as housing facilities. This proved to be highly challenging, and was commonly declined by individual service managers due to limited resources:

The caseworker asked whether we could provide physiotherapy in the suggested facility. We don’t have the resources to do that. (Interview, manager of local municipal physiotherapy unit)

Commonly, the final decisions regarding the location of municipal rehabilitation overruled care providers’ professional judgements:

They (the municipal rehabilitation unit) reported that it was difficult for them to receive cognitively impaired individuals. And then there were also discussions that this was a person who needed one-to-one follow-up . . . So, there was a lot of back and forth. We discussed it at the decision-making office. And I guess we all agreed that the only institutional offer we could provide was at that rehabilitation unit. We have no other offer. Then we had some discussions with the municipal rehabilitation unit, but we stated that this is what we have to offer. (Interview, caseworker)

Our analysis showed recurring adherence to guidelines, with limited flexibility among the caseworkers to adapt services to professional recommendations or individual preferences. It was stressed that deviations would require the relocation of both personnel and financial resources from other areas of the municipal services and that this would jeopardise the goal of fair and equal treatment overall. However, in 2 cases deviations from the local guidelines occurred. In one case, recommendations from municipal care providers were followed in the initial stages of discharge planning, and considerable additional resources were allocated with reference to the complex needs of the patient. In the second case, the deviation entailed a prolonged stay at the hospital paid for by the municipality. Following the prolonged hospital stay, the patient was assigned to the original location designated by caseworkers, although initially disputed by both next of kin and hospital staff. Our data does not describe the reasoning behind this deviation, but both the deviations described above involved higher top- executive managers at the municipal level.

## Discussion

In this study, we explored the practice of transitions and service allocation for rehabilitation patients with moderate or severe brain injury. A major concern for all involved was the location for further municipal rehabilitation. Through our analysis the intricate path towards a decision on service allocation became visible. The municipal caseworkers had the arduous task of navigating the processes of gathering and managing information leading to a decision.

Our analysis showed that the transition process took considerable time and was prepared weeks and months ahead of the hospital discharge. This seemed in contrast with existing studies, which report time pressure and a need for quick decisions and solutions,^14,28,41,42^ but is in accordance with suggestions regarding an early-initiated dialogue about discharge plans between stakeholders.^57^ Based on our data, we cannot draw conclusions of whether caseworkers experienced time pressure. An explanation for our results could be that the patients in our study had complex needs and were discharged from a rehabilitation unit where they had spent considerable time following treatment in an acute ward. Our findings seem to underline that for patients with complex needs, transition planning needs to address the decision of service allocation as a distinct, important and potentially time-consuming process.

Despite the long-term planning, the process of service allocation was challenging due to discrepancies between service providers’ recommendations and preferences and the needs of patients and next of kin, and organisational requirements stated in local guidelines and the organisation of services. These findings resonate with previous research that points to the challenges of balancing aspects of individually tailored services and organisational restraints in service allocation impacting care transitions.^14,22,38,41,42,47^ The challenges highlight the crucial role of caseworkers in the service allocation process.

It is suggested that to incorporate person-centred care, where services are adjusted to individual needs and preferences and where patients and next of kin participate and impact decisions of their care trajectory, discretionary space is required.^22,58^ Our findings showed that the caseworkers invested considerable time finding solutions in order to take a discretionary approach to fulfil the recommendations of health care providers, and the preferences and needs of patients and next of kin. This finding corresponds to the street-level literature describing discretionary space as a prerequisite for service allocation to facilitate individually tailored services adjusted to individual needs and preferences.^39^ However, our data also revealed that the final decisions were often in accordance with the solutions foreshadowed during the initial meetings, although disputed by next of kin and healthcare providers. This suggests a highly limited discretionary space for the caseworkers, where decisions were predetermined and corresponded to municipal allocation guidelines, providing little flexibility to make deviating choices. Our data indicated that this is a great hindrance to achieving the goal of user involvement to the extent where the individual needs and preferences of the patient and next of kin could impact the decision of service allocation. Also, earlier research reports that organisational restraints impose stronger obligations than caseworkers’ liberty to opt for discretional judgements and individually tailored services.^14,38,41,45^ Furthermore, our results seemed in accordance with the intentions of the purchaser-provider model, with standardisation of service allocation. However, it also illuminated how the standardisation of the service allocation process may interfere with the caseworker’s possibility to provide tailored services to individual needs and preferences, undermining the possibility of variation.^59^

Our analysis illustrated that the caseworkers were in a precarious position when representing the municipality, facing tensions when the intensity of specialist services was to be downscaled while the patients still needed comprehensive rehabilitation services. Our observations suggested that caseworkers applied different strategies to handle the tensions, which have been reported previously.^38,41^ These included adherence to the local guidelines, discussions within peer meetings, decision-making separated from patients and health care providers and contacting superiors to elaborate on services already decided on. This study showed that recommendations from care providers (both specialist and municipal care providers) regarding potential positive outcomes were undermined and even abandoned in favour of meeting municipal regulations. Moreover, patients or next of kin could have requests and needs that were incompatible with what the municipality had to offer. One argument in favour of implementing the purchaser-provider model was the need to separate decision-making authority from care providers, to facilitate more neutral decisions, while maintaining an overview of total available resources and needs in the municipality.^37,59^ Our data revealed that comprehensive challenges and dilemmas may still arise as decisions are made between conflicting requirements, such as needs versus resources. That is, as decisions needed to be made while contrasting options did not provide satisfactory solutions as requirements clashed with one another.^60^ If the caseworker had accommodated the recommendations of healthcare providers and requests of patients and next of kin, this would have broken with municipal guidelines. However, as the caseworker applied guideline criteria, the requirement of individually tailored services remained unmet.

Tensions of street-level work by caseworkers became particularly evident at the joint meetings where patients and next of kin could discuss specific issues regarding their services. Some authors have discussed that meetings that are thought to empower patients/next of kin through their involvement, may have the opposite effect.^40,47^ The involvement might rather become proforma if patients or next of kin experience difficulties in wording their expectations or if decisions are difficult to influence.^47^ Furthermore, the tensions at the meetings may have impacted further cooperation and trust, and substantiated the notion of a fragmented healthcare service among patients and next of kin.^24^

The involvement of patients and next of kin is comprised in the concept of person-centred care which is well promoted in health and social care.^6,24^ Our findings reveal that despite caseworkers strive to include a person-centred approach by asking about preferences and needs, such questions seemed hardly answerable for patients or next of kin. Our approach did not focus on what restrained participants from answering, but explanations may relate to other authors’ findings showing that patients and next of kin can feel overwhelmed, uncertain and powerless.^61^ Recent literature reports strategies that facilitate good transitions, discharge planning and shared decision making. These include communication strategies between all stakeholders within and between organisations and family members, earlier goal setting to develop shared understanding and expectations or provision of education to increase understanding and communication.^57^ From a street level perspective, the question remains as to how person-centred care can be achieved in relation to the demands of organisational guidelines to ensure just and equal treatment within resource limitations.

As in other studies, we found that specialist healthcare providers in several instances portrayed services for the patients that were unavailable in the municipalities.^46,62^ It has been argued that such discrepancies appear due to a lack of knowledge or understanding of other parts of the healthcare system apart from their own.^15,63^ However, our study indicates that the hospital staff continued to negotiate alternative solutions despite being informed that the recommended services were unavailable. Our findings might be an indication, as previously shown, that healthcare providers who experience being overruled on their expert advice, take on an advocating role.^54^ Alternatively, the findings may be an indication that the hospital staff has an expectation of a more specialised health care at the municipal level, disregarding municipal resource limitations. This could be related to the implementation of the Norwegian Coordination reform creating a shift in the notion and execution of healthcare services in general and more specifically in rehabilitation.^36,46,62,64,65^ More research is needed to examine this issue further. Caseworkers suggested meetings with hospital staff to coordinate efforts prior to the involvement of patients and next of kin. Although this might possibly conceal open disagreement about services and give an impression of a coordinated health service, it directly contradicts transparency and shared decision-making. Yet, the organisation of meetings should be investigated for further improvement. As common meeting points are deemed to be of great importance to ensure knowledge transfer and improve the transition of patients, the latter might be imperative for enhancing care transitions.^46^

The findings of this study support earlier reports of service allocation being based on the availability and standardisation of services, rather than individual needs or preferences.^14,46^ At the same time, the results support earlier findings showing that healthcare providers and caseworkers tried to find solutions, although these were limited.^46^ Hence, this study supports previous findings of caseworkers striving to strike a balance between conflicting demands.^38^ It is important to note that we found that 2 transition decisions deviated from the guidelines and the organisational structure. For these transitions, the decisions involved a higher level of executive involvement. This indicated that there was discretionary space within the municipal organisations to facilitate individually tailored services, but that the caseworkers did not hold the decision-making power to relocate resources.

## Methodological Considerations and Limitations

This qualitative study followed real-time transitions and illustrated the challenges of ongoing processes of service allocation within such transitions. To enhance validity, member checking and triangulation of data sources and investigators were employed.^55^ The study emphasised written documents from patient journals to create a chronological overview of individual transitions from hospital to municipal rehabilitation, focusing on service allocation. By integrating these documents with interviews and observational data, a comprehensive database of individual trajectories and overall data was developed, facilitating comparison between trajectories and strengthening the study’s reliability.^49^ The pre-understanding and practical experience from the field of rehabilitation influenced the development of the study design. The in-depth understanding of the rehabilitation context may have facilitated the data collection process. The researchers reflected over the challenges attached to their positions. In order to enhance reflexivity, the study was discussed with experienced researchers from different fields in all phases. The involvement and discussions in the analytical process between the 3 authors, from diverse clinical and academic backgrounds, enhanced the reflexivity of the study.^55^ Member checking allowed us to challenge the data gathered and interpretations, increasing the study’s credibility.^55^

The study has some limitations worth noting. The study’s design is inherently limited in scope, which may limit transferability to other contexts. This study provides insights into descriptions of service allocation for ABI-patients in Norway. Due to the detailed description of the study’s context, others can transfer the results to similar contexts. While unique characteristics of the context exist, the study still addresses broader issues applicable to similar settings. Different research approaches may have provided different interpretations and results. Additional data, including interviews with hospital personnel, could have provided further insights into the service allocation process. In addition, a deeper exploration of the interviews could have provided further insights into the clinical reasoning and experiences of patients and next of kin.

## Conclusions

The service allocation process for the studied transitions proved to be highly demanding due to discrepancies between service providers’ recommendations, the preferences of patients and next of kin and organisational requirements stated in the local guidelines and the organisation of services. The study showed that the caseworkers adhered to the allocation guidelines, despite the complexity of needs, suggesting that deviations were difficult to make. Hence, this study indicates that incorporating individual tailored services into the daily service allocation practice is highly challenging, despite the recognition of the importance of user involvement and coordination of services. This knowledge is important to inform the practice field and policies for individuals with complex rehabilitation needs, to ease the transition from hospital to municipal rehabilitation. Though the data is gathered within the Norwegian healthcare setting, our findings may also be relevant elsewhere, suited to contribute to the ongoing discussions and efforts to improve care trajectories internationally. Research on transitions and service allocation decisions within different contexts could provide important data to compare and contrast the findings presented in this study. Further, questions of how to effectively design guidelines and transition processes while supporting the different stakeholders throughout the challenges of the transition process remain.
